# Cytotoxicity of Different Nano Composite Resins on Human Gingival and Periodontal Ligament Fibroblast Cell Lines: An In Vitro Study

**DOI:** 10.3390/biomedicines8030048

**Published:** 2020-03-01

**Authors:** Gamze Kavuncu, Ayse Mine Yilmaz, Betul Karademir Yilmaz, Pinar Yilmaz Atali, Elif Cigdem Altunok, Leyla Kuru, Omer Birkan Agrali

**Affiliations:** 1 Department of Periodontology, Faculty of Dentistry, Marmara University, Istanbul 34854, Turkey; gamzekavuncu1990@gmail.com (G.K.); lkuru@marmara.edu.tr (L.K.); 2 Department of Biochemistry, Faculty of Medicine, Marmara University, Istanbul 34854, Turkey; aysemine.yilmaz@gmail.com (A.M.Y.); btlkarademir@gmail.com (B.K.Y.); 3 Genetic and Metabolic Diseases Research and Investigation Center, Marmara University, Istanbul 34854, Turkey; 4 Department of Restorative Dentistry, Faculty of Dentistry, Marmara University, Istanbul 34854, Turkey; dtpinaryilmaz@gmail.com; 5 Department of Biostatistics and Medical Informatics, Faculty of Medicine, Yeditepe University, Istanbul 34755, Turkey; ecaltunok@yeditepe.edu.tr

**Keywords:** biocompatibility, cell culture, composite resin, cytotoxicity, fibroblasts

## Abstract

The aim of this study is to determine the cytotoxicity of three different nano composite resins (CRs) on human gingival fibroblast (hGF) and periodontal ligament fibroblast (hPDLF) cell lines. These CRs selected were nanohybrid organic monomer-based Admira Fusion (AF), nanohybrid Bis-(acryloyloxymethyl) tricyclo [5.2.1.0.sup.2,6] decane-based Charisma Topaz (CT), and supra nano filled resin-based Estelite Quick Sigma (EQS). MTT assay was performed to assess the cytotoxicity of CRs at 24 h and one week. AF and EQS applied on hGF cells at 24 h and one week demonstrated similar cytotoxic outcomes. Cytotoxicity of CT on hGF cells at one week was higher than 24 h (*p* = 0.04). Cytotoxicity of CT on hGF cells was higher at 24 h (*p* = 0.002) and one week (*p* = 0.009) compared to control. All composites showed higher cytotoxicity on hPDLF cells at one week than the 24 h (AF; *p* = 0.02, CT; *p* = 0.02, EQS; *p* = 0.04). AF and EQS demonstrated lower cytotoxicity on hPDLF cells than the control group at 24 h (AF; *p* = 0.01, EQS; *p* = 0.001). CT was found more cytotoxic on hPDLF cells than the control (*p* = 0.01) and EQS group (*p* = 0.008) at one week. The cytotoxicity of CRs on hGF and hPDLF cells vary, according to the type of composites, cell types, and exposure time.

## 1. Introduction

Dental restorations are widely used to repair the function and aesthetics by ensuring tooth surface integrity. For this purpose, materials have been developed to mimic the physical and morphological characteristics of teeth; yet, the ideal material has not been discovered. 

Composite resins (CRs) are still the most common used dental restorative material in dental practice and revolutionized restorative dentistry in the mid-20th century. In addition to their aesthetic success, some of the advantages include stability in the oral environment and ease of application. On the other hand, resin-based composites possess disadvantages such as polymerization shrinkage, the risk of secondary caries due to unsuccessful connection between resin and dentin, high thermal expansion coefficient and low wear resistance compared to metal-based restorations. To overcome these disadvantages, the structure of the CRs has been improved by changing their filler amount, size, shape, monomer type, and modifying initiators for the polymerization reactions. Besides improvements in filler systems, such as supra nano particles, nanohybrid CRs showed a variation in monomer–matrix formulations. Instead of bisphenol-A diglycidyl ether dimethacrylate (Bis-GMA), bisphenol A polyethylene glycol diether dimethacrylate (BIS-EMA), urethane dimethacrylate (UDMA), or Triethylene- glycol dimethacrylate (TEGDMA), newly developed monomers either replaced these monomers or only added in a resin structure [1]. Ormocer (organically modified ceramic) and Bis-(acryloyloxymethyl) tricyclo [5.2.1.0.sup.2,6] decane (TCD-DI-HEA) monomers are the alternatives of the traditional monomers used in the resin structure of the resin-based composites [2]. Ormocer composites are free of dimethacrylate monomers. These have either no or minimal cytotoxicity matters compared to conventional resin based composites [2]. TCD-DI-HEA monomer have two major properties as being a low-shrinkage methacrylate and biocompatible monomer [3]. This is important because the periodontal-restorative relationship is frequently observed clinically near or under the cervical and proximal margins of the restorations [4,5,6]. When the margin of the CR restorations extended to gingival or subgingival areas, long-term clinical success may be questionable [7,8,9,10,11]. Studies have shown that dental restorations in contact with periodontal tissues may lead to gingival inflammation and periodontal attachment loss [12,13,14]. It has been reported that the use of some CRs in the subgingival area may cause gingivitis [15,16]. Furthermore, it has been speculated that unpolymerized monomers leaking from composites may be harmful to surrounding soft tissues [17,18]. In order to asses this phenomenon, several studies took place investigating any possible negative upshot on the cells/tissues [17,18,19,20,21,22,23,24]. 

One of the prominent methods in the studies assessing the biocompatibility of CRs is the evaluation of cytotoxicity and other influences of these materials on cell lines in vitro [20,21,22]. The most important issue in the cytotoxicity studies of composites was the impact of composite forming monomers released during the polymerization [19,23,24]. Even after polymerization, monomers and their chemical degradation products affect long-term stability and biocompatibility of CRs [19,23,25]. In vitro studies have shown that composite components can yield cytotoxic, genotoxic, mutagenic, and estrogenic effects via influencing the cell response resulting in apoptosis or cell cycle delay. [26,27,28]. Schubert et al. [29] evaluated the cytotoxic effects of nanohybrid composite, nanohybrid ormocer, and nanophilic composites on mouse L929 fibroblast and human gingival fibroblast (hGF) cells for 48 and 72 h. At the end of 48 or 72 h incubation time, the ormocer composite group exhibited the lowest cytotoxic effect on L929 fibroblast and hGF cells. Moreover, the evaluation of the effects of incubation time on cell viability showed that the cytotoxic effect of ormocer composites decreased significantly in L929 fibroblast cells and the cytotoxicity effect of nanohybrid and nanofiller composites increased over time [29]. Yang et al. [30] investigated the monomers released from five different CRs and their cytotoxic and genotoxic impressions on hGF cells after 72 h incubation period. They found that monomers, such as 2-hydroxyethyl methacrylate (HEMA), TEGDMA, ethylene glycol dimethacrylate (EGDMA) were present with high concentrations in composite eluates. On the other hand, released monomers had no significant cytotoxic effect on hGF cells [30]. Furthermore, DNA double chain breakage was established by the compounds released from two different Bis-GMA containing micro-hybrid composites [30]. 

Under the light of these findings, the aim of this study is to evaluate the in vitro cytotoxicity effect of three different monomer-based nano CRs on hGF and human periodontal ligament fibroblast (hPDLF) cell lines for 24 h and one week time periods. The null hypothesis was that the use of novel nano CRs with different monomer ingredients (including Bis-GMA, Ormocer or TCD-DI-HEA) would not influence their cytotoxicity behavior, considering the periodontal-restorative relationship.

## 2. Materials and Methods

The three materials investigated in this study were nanohybrid ormocer Admira Fusion (AF) (Nano-Hybrid-Ormocer, Admira^®^ Fusion, VOCO GmbH, Cuxhaven, Germany), nanohybrid Bis-(akryloyloxymethyl) tricyclo [5.2.1.0.sup.2,6] decane-based Charisma Topaz (CT) (Nano-Hybrid Composite Charizma^®^ Topaz, Heraeus Kulzer, Wehrheim, Germany), and resin-based Estelite Quick Sigma (EQS) (Supra-Nano-Fill Resin Composite, Estelite^®^ Σ Quick Sigma, Tokuyama Dental, Tokyo, Japan). 

### 2.1. Preparation of Material Samples

Composite disc samples with a diameter of 7 mm and a height of 2 mm were prepared according to ISO 10993-12:2012 standards by using customized molds and consistent with the manufacturers’ instructions [31,32]. While condensation of the unpolymerized composite was achieved on a glass plate, mylar matrix strip was applied on the surface to limit oxygen inhibition. Polymerization was accomplished using a LED-light source (VALO^®^, Cordless Curing Light; Ultradent, USA) at an average 720 mW/cm^2^ for 20 s applied to both bottom and top surfaces of the disc. Then, the composite disc samples (*n* = 3) were UV sterilized prior to cytotoxicity test.

### 2.2. Cell Culture

The hGF (ATCC, CRl-2014, Manassas, VA, USA) and hPDLF (LONZA, CC7049, Walkersville, MD, USA) cell lines were maintained in Dulbecco’s Modified Eagles Medium (DMEM) (Pan-Biotech, Germany), supplemented with 500 U/mL penicillin and 50 µg/mL streptomycine at 37 °C in humidified chamber with 5% CO_2_. The cells were allowed to grow in 175 cm^2^ culture flasks. After reaching the confluence of approximately 70%–80%, the cells were washed with phosphate buffered saline (PBS) (LONZA, Sweden) and detached from the flasks by a brief treatment with trypsin/EDTA (Pan-Biotech, Germany). Approximately 1 × 10^5^ cells were seeded per well of 24-well cell culture plate in 2 mL of growth medium. Cells were incubated at 37 °C and 5% CO_2_ and 90% humidity for 24 h until the cytotoxicity test.

### 2.3. Cytotoxicity Test

One composite disc sample was placed on each well (*n* = 3). The ratio of cell layer surface area in contact with the sample surface area was measured as 18,15%. Then, the cells were seeded and incubated in direct contact with composite disc samples at 37 °C and 5% CO_2_ and 90% humidity for 24 h and one week. The wells without composite disc sample were served as negative control group, whereas the wells with different composite disc samples were considered as the test groups. After the incubation period, composite disc samples were removed and the culture medium was discarded. Cytotoxicity and cell viability tests were performed by using 3-(4,5-Dimethylthiazol-2-yl)-2,5-Diphenyltetrazolium Bromide (MTT) based on the mitochondrial dehydrogenase activity [31]. Confluent cells were washed three times with PBS and 66 µL MTT solution (1 mg/10 mL in PBS) was added. After 2 h incubation, 1 mL solubilizing buffer (10 g SDS/99.4 DMSO + 0.6 mL acetic acid) was added per well and 100 µL medium from each well was transferred to two wells of 96-well plate. Absorbance was measured with a plate reader (Perkin Elmer, Boston, USA) at 590 nm (*n* = 6).

### 2.4. Statistical Analysis

The cytotoxicity and viability of cells were calculated according to the control group, which had a cytotoxicity volume of 0% and viability volume of 100%. Data were analyzed by using SPSS statistical package (SPSS 25.0, IBM Inc., Armonk, NY, USA). Non-parametric statistical methods were used for the statistical analysis of the data, which were not normally distributed and were shown in detail with median, minimum, maximum, mean, and standard deviation values. The Wilcoxon test was used for intragroup comparison, Kruskal Wallis for intergroup multiple comparisons. The Mann Whitney-U with post hoc Bonferroni test was performed for paired comparison. Significance value was set as *p* < 0.05.

## 3. Results

Cytotoxicity and viability assessment revealed cell type-, composite material type- and time-dependent distinctive results. In this regard, AF and EQS CRs displayed no cytotoxic effect on the hGF cells at 24 h compared to the control group (*p* > 0.05) (Table 1).

However, CT was cytotoxic on hGF cells at 24 h compared to the control group (*p* = 0.002) (Table 1). In parallel with this finding, the viability of hGF cells in the AF and EQS groups were similar to the control groups (*p* > 0.05) (Table 2).

**Figure 1:**
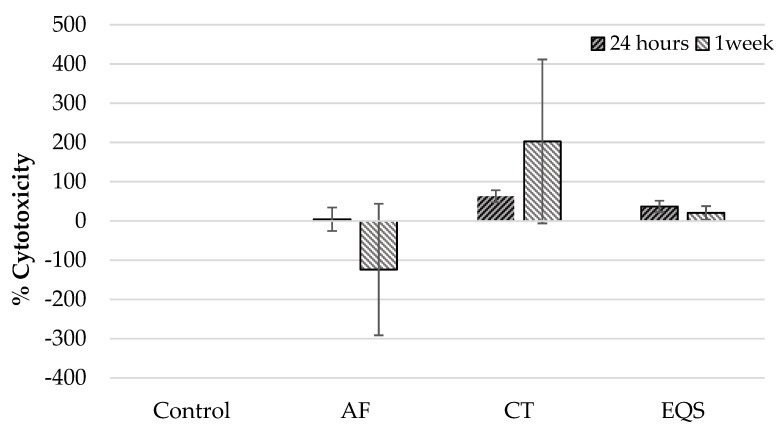
Cytotoxicity of CRs on hGF cells at 24 h and one-week time periods (mean ± SD).

In addition, the viability of hGF cells in the CT group was lower than the control group at 24 h (*p* = 0.002) (Table 2). Besides, one-week cytotoxic effects on hGF cells were similar to 24 h for all CRs. According to this, AF and EQS showed no cytotoxicity on the hGF cells compared to the control group at one week (*p* > 0.05) (Table 1). However, CT was cytotoxic on hGF cells at one week in comparison to the control group (*p* = 0.009) (Table 1). Moreover, paired comparisons revealed a lower cytotoxic impact of the AF group on hGF cells compared to the effects of the CT (*p* = 0.03) and EQS (*p* = 0.00) groups at one week (Table 1). The viability of hGF cells at one week for each group has supported cytotoxicity results. Thus, the viability of hGF cells in the AF and EQS groups was similar to the control group (*p* > 0.05) (Table 2), but for the CT group, it was significantly lower than the control group (*p* = 0.009) (Table 2). Comparisons of cytotoxicity (Table 3) (Figure 1) and viability (Table 4) findings for each experimental group between one week and 24 h have demonstrated that only CT was more cytotoxic on hGF cells at one week than 24 h (*p* = 0.04).

**Figure 2:**
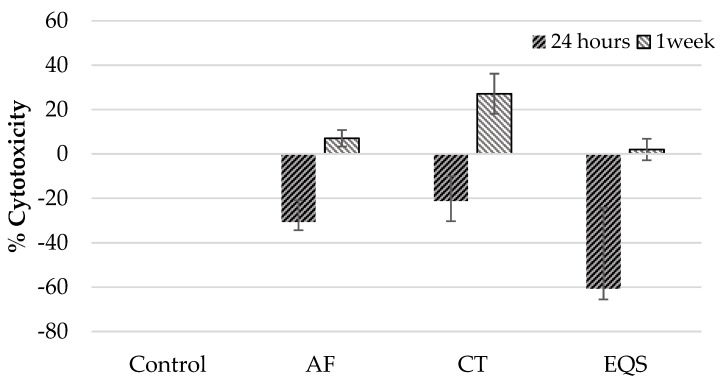
Cytotoxicity of CRs on hPDLF cells at 24 h and one-week time periods (mean ± SD).

Evaluation of the cytotoxicity and cell viability effects of the CRs on hPDLF cells at 24 h and one week time periods revealed slightly different results compared to the outcomes on hGF cells. CT composite material was not significantly cytotoxic on hPDLF cells at 24 h compared to the control group (*p* > 0.05) (Table 1). Meanwhile, in paired comparisons, the cytotoxicity of the AF and EQS CRs on hPDLF cells was significantly less than the control group at 24 h (AF; *p* = 0.01, EQS; *p* = 0.001) (Table 1). In the one week cytotoxicity assessment of CRs, only CT was cytotoxic on hPDLF cells (*p* = 0.01) (Table 1). In addition, the cytotoxicity effect of CT on hPDLF cells at one week was higher than that of EQS (*p* = 0.008) (Table 1). Viability of hPDLF cells at one week was lower in the CT group than the control group (*p* = 0.001) (Table 2). All CRs demonstrated higher cytotoxicity on hPDLF cells at one week compared to 24 h (AF; *p* = 0.02, CT; *p* = 0.02, EQS; *p* = 0.04) (Table 3) (Figure 2).

Accordingly, viabilities of hPDLF cells in contact with AF, CT, and EQS composite discs were lower at one week compared to 24 h (AF; *p* = 0.02, CT; *p* = 0.02, EQS; *p* = 0.04) (Table 4).

## 4. Discussion

The importance of periodontal-restorative relationship surges in cases of severe tooth loss, deep caries, and abrasive defects of the root surface observed after gingival recessions. Therefore, this relationship has been the subject of various investigations [33] evaluating the effect of restoration boundary location [13,16], surface properties [15,33], and restoration type [16,20,34,35,36] on periodontal tissues. Nowadays, ever-increasing aesthetic demands from patients and recent developments in restorative materials have raised the prominence of research assessing the effects of materials on periodontal tissues. Studies have shown that restorations can produce an inflammatory response in soft tissues adjacent to the restorative material [10,15,36]. Recently, the increase in the use of nanotechnological methods in composite construction led to the production of new generation nanohybrid composites, resulting in improvements in the physical properties of composites such as surface smoothness, mechanical durability, and light transmission. This in vitro study is the first study to investigate the cytotoxicity of three different nano CRs with different monomer contents such as AF, EQS, and CT on hGF and hPDLF cell lines.

The principle of in vitro methods used for examining the harmful effects of materials in biological tissues is based on monitoring the molecular and cellular changes that occur in the cell as a result of contact with the material [31]. In vitro methods assessing the biocompatibility of materials were classified as; “direct contact test”, which is based on direct contact of the material with the cell; “indirect contact test” includes an intermediate layer, such as agar gel or Millipore filter between the material and the cell, and “extract test”, which means the application of eluates released from the material to the cells [31,37]. It has been stated in many studies that the direct contact test is a method that provides more sensitive results in the determination of material toxicity, compared to the other methods [38,39]. In our study, composite disc samples were brought into contact with the cells in accordance with the direct contact test method and kept for either 24 h or one week time periods.

Cytotoxicity and cell viability measurements are used in many different fields in a broad range spectrum. Cytotoxicity is related to the cell lining parameters, cell membrane integrity, cell volume, cytoplasmic volume (that is, shrinkage) refractive index of the cell, propensity to cleave DNA and related nuclear condensation. Cell membrane properties depend on parameters such as lipid layer integrity, which would depend on octanol partition coefficients/hydrophobicity. More insights into molecular mechanisms of cytotoxicity can be obtained from statistical and neural network analysis [40]. An ideal assay for in vitro should not interfere with the test compound and may change the interpretation of compound interaction. Viability/cytotoxicity assays should be selected according to the cell type and the properties of the substance to be measured. Since luminescence and fluorescence methods are particularly sensitive, they are more likely to cause interference when examining the effects of substances. Therefore, spectrophotometric methods may be preferred in these circumstances [41]. The use of colorimetric MTT test for the evaluation of cytotoxicity in cells was preferred in the present study due to the fact that it delivers rapid and objective results, as well as provides information on viability via cell metabolic activity [42,43,44].

Cell-based studies evaluating the effects CRs on surrounding tissues were focused on the cytotoxic effects of monomers released after polymerization [3,24,44]. In in vitro cytotoxicity tests for composites, the ratio between sample surface area and culture medium volume was reported to have an impact on the outcome [37]. From a clinical point of view, the mean surface area of mesial-occlusal-distal fillings was calculated as 95 mm^2^, in cervical fillings as 12 mm^2^, and in veneer restorations as 86 mm^2^ [45]. In our study, composite discs were prepared with 7 mm diameter and 2 mm thickness [31,32]. Thus, the surface area of each disc was calculated as 120.89 mm^2^ considering the relationship between the average surface area of the fillings and the releasable monomers. Moreover, considering the ISO 10993-5 guideline for standardization, it was suggested that the ratio of the cell-composite disc contact surface area to the well surface area should be at least 10% [46]. In the present study, the ratio of the cell contact surface of the composite disc to the well surface area was calculated as 18.15% in accordance with ISO standards.

In cytotoxicity studies, continuous cell lines, such as 3T3 and L929 mouse fibroblasts, are particularly favored because of their biological response, ease of production, and easy control of their culture [17,47]. However, it has been reported that although working with target tissue primary cells in the laboratory may have some difficulties such as shorter life span and slower growth of the cells, using the primary cells of the related tissue would provide more meaningful results in order to consider the clinical relationship [17,22,28,47]. In our study, the hGF and hPDLF cell lines were used in order to mimic the clinical relationship with the in vitro environment.

Despite the increased use of nanohybrid composites worldwide, the studies investigating the impacts of these materials on surrounding tissues with their long-term exposure times are limited. On the other hand, several studies suggest that different composites may possess different cytotoxic effects, albeit, the findings are somewhat inconsistent [18,30,48,49]. Compared to the traditional CRs, ormocer composites were reported to produce less monomer release and show less cytotoxicity [29,30,42,50]. Yang et al. [30] evaluated 72 h monomer release of five different CRs in DMEM together with the cytotoxicity of their released monomers on hGF cells, and they reported that the ormocer group composite material did not alter cell viability. In our study, the lowest cytotoxicity on hGF cells was observed in the ormocer AF group in both 24 h and 1-week intervals (*p* < 0.05). This finding is consistent with previous studies regarding decreased cytotoxicity of ormocers related to the less monomer release deceptively leading enhanced biocompatibility compared to resin-based dental restorative materials [29,30,37]. CT differs from other CRs with its new generation monomer content. Its monomer elution has been evaluated in a limited number of studies demonstrating a continuous release after an incubation period of a few weeks [3,51,52]. In our study, CT was detected as the most toxic material among three for hGF cell line at 24 h and 1-week time periods. TCD-DI-HEA monomer and its amount of release may be suggested as the reasons for this result. The impacts of this monomer on the human cell behavior such as viability, growth, and immune responsivity are not yet fully understood. Further studies having longer evaluation periods are needed to investigate the in vitro effects of this monomer on human cell lines because long-term elution and ensuing chronic exposure to monomers from resin based dental materials should not be ignored considering the human health risks [3].

Developing new properties of composites bring forward clinical studies investigating the success of subgingival restorations and restorative materials that can be also used during periodontal surgeries [5,10,15]. Therefore, the close relationship between the restorative materials and the periodontal ligament cells in the subgingival area needs to be further investigated. To our knowledge, in the only study evaluating the influences of EQS and various endodontic cements on hPDLF cells, no significant change was observed in the cell count on the first day, but the number of cells in the EQS composite group decreased significantly on the eighth day [53]. However, so far, there is no study assessing the effects of AF and CT CRs on hPDLF cells. In our study, we evaluated the cytotoxic effects of three different CRs on hPDLF cells, but no significant cytotoxicity was observed in any of the composite groups within 24 h, except the CT group at one week. This result may be related to the short-term resistance of hPDLF cells to the possible cytotoxic effect of materials [22]. It has been suggested that cytotoxicity increases as the duration of exposure increases in relation to the cumulative effect caused by continuous monomer release from the materials [3]. Schulz et al. [49] examined the effects of different CRs on gene expression of hGF and human gingival keratinocyte cells in 24 h and one week intervals and indicated that cytotoxic effect on hGF cells increased in a one-week period. Anand et al. [42] concluded that human osteoblast cells are more sensitive to exposure time regardless of dose. In the present study, it was observed that the cytotoxicity of the CT group on hGF cells increased in time as well as the cytotoxicity of all composite groups on hPDLF cells; however, none of the composite groups reached the lethal dose.

Lack of the observations investigating the cytotoxic effects of the individual monomer ingredients of the CRs conducted at further different time periods out of 24 h and one-week intervals may be considered as the limitations of the study. On the other hand, an overview can be achieved from our study for the early and late term biocompatibility of the recently launched nano dental CRs. Currently, there is limited number of studies evaluating the cytotoxicity impact of these dental nano CRs on human periodontal cells [22,29,30,42,49,50,53]. The current study has a prominence to shed light on the subject in this respect, but future studies are needed to evaluate the long-term monomer elution of nano CRs and their biocompatibility with human periodontal cells.

## 5. Conclusions

The fact that AF does not show a cytotoxic effect on hGF and hPDLF cells may suggest that the ormocer group composites can be considered as biocompatible in clinical cases that require restoration in close association with the gingiva and periodontal ligament. It can be concluded that Bis-GMA and TEGDMA containing supra-nano-hybrid resin-based composite material EQS has no cytotoxic effect on hGF and hPDLF cells, probably due to its enhanced polymerization technology leading to the reduction of monomer release. Further studies are warranted to investigate the effects of a new TCD-DI-HEA monomer containing CT on human periodontium cells due to its cytotoxic impact on the hGF and hPDLF cells. In addition to the studies demonstrating that different cell types show different levels of biocompatibility reactions to the similar stimuli and time parameters, within the limits of this study, we may suggest that the type of the composite resin matters by means of cytotoxic influence under the same time and cell type circumstances. On the other hand, time parameters may not be a game changer in the biocompatibility of a resin composite applied to the same cell type.

## Figures and Tables

**Table 1:** Intergroup and paired cytotoxicity comparisons of composite resins (CRs) on hGF and hPDLF cell lines at 24 h and one week time periods.

Cell/Time	% Cytotoxicity Groups (*n*= 6) Median Min–Max (Mean ± SD)				Paired Comparisons						
	Con ^a^	AF ^b^	CT ^c^	EQS ^d^	*p* ^*^	*p* ^#(a,b)^	*p* ^#(a–c)^	*p* ^#(a–d)^	*p* ^#(b,c)^	*p* ^#(b–d)^	*p* ^#(c,d)^
hGF/24 h	0.00 0.00–0.00 (0.00 ± 0.00)	20.95 −38.09–30.20 (4.53 ± 32.77)	67.29 39.92–78.82 (62.91 ± 6.58)	46.14 15.01–48.78 (37.12 ± 15.75)	0.001	1.00	0.002	0.08	0.031	0.59	1.00
hGF/one week	0.00 0.00–0.00 (0.00 ± 0.00)	−69.44 −488.09–1.09 (123.77 ± 183.63)	101.78 48.61–655.55 (202.67 ± 228.76)	17.36 4.16–55.55 (20.69 ± 19.02)	0.001	1.00	0.009	0.46	0.00	0.03	0.97
hPDLF/24 h	0.00 0.00–0.00 (0.00 ± 0.00)	−28.73 −50.00–−18.96 (-21.73 ± 10.66)	−22.91 −38.18–−1.56 (-21.23 ± 12.25)	−44.44 −123.63–−20.31 (-60.70 ± 40.76)	0.000	0.01	0.17	0.001	1.00	1.00	0.63
hPDLF/one week	0.00 0.00–0.00 (0.00 ± 0.00)	6.17 −1.76–12.29 (7.02 ± 4.06)	31.02 13.27–37.90 (27.10 ± 9.88)	2.86 −6.36–6.89 (1.97 ± 5.29)	0.001	0.2	0.01	1.00	0.3	1.00	0.008

* Kruskal Wallis test, *p* ˂ 0.01, ^#^ Mann Whitney-U test with Bonferroni correction, hGF: Human Gingival Fibroblast, hPDLF: Human Periodontal Ligament Fibroblast, ^a^ Control, ^b^: Admira^®^ Fusion, ^c^: Charizma^®^ Topaz, ^d^: Estelite^®^ Σ Quick Sigma, SD: Standard Deviation.

**Table 2:** Intergroup and paired viability comparisons of CRs on hGF and hPDLF cell lines at 24 h and one week time periods.

Cell/Time	% Viability Groups (*n*= 6) Median Min–Max (Mean ±SD)				Paired Comparisons						
	Con ^a^	AF ^b^	CT ^c^	EQS ^d^	*p* ^*^	*p* ^#(a,b)^	*p* ^#(a–c)^	*p* ^#(a–d)^	*p* ^#(b,c)^	*p* ^#(b–d)^	*p* ^#(c,d)^
hGF/ 24 h	100 100–100 (100 ± 0.00)	79.04 69.79–138.09 (95.46 ± 32.77)	32.70 21.17–60.07 (37.06 ± 16.58)	53.85 51.21–84.98 (62.87 ± 15.75)	0.001	1.00	0.002	0.08	0.031	0.59	1.00
hGF/ one week	100 100–100 (100 ± 0.00)	169.44 98.90–588.88 (223.77 ± 183.63)	−1.78 −555.55–51.38 (−102.67 ± 228.76)	82.63 44.44–95.83 (79.30 ± 19.02)	0.000	1.00	0.009	0.46	0.00	0.03	0.97
hPDLF/ 24 h	100 100–100 (100 ± 0.00)	128.73 118.96–150.00 (130.62 ± 10.66)	122.91 101.56–138.18 (121.23 ± 12.25)	144.44 120.31–223.63 (160.70 ± 40.76)	0.01	0.01	0.17	0.001	1.00	1.00	0.63
hPDLF/ one week	100 100–100 (100 ± 0.00)	93.82 87.70–98.23 (92.97 ± 4.06)	68.97 62.09–86.72 (72.89 ± 9.88)	97.13 93.10–106.36 (98.02 ± 5.29)	0.001	0.2	0.001	1.00	0.36	1.00	0.008

* Kruskal Wallis test, ^#^ Mann Whitney-U test with Bonferroni correction, hGF: Human Gingival Fibroblast, hPDLF: Human Periodontal Ligament Fibroblast, ^a^ Control, ^b^: Admira^®^ Fusion, ^c^: Charizma^®^ Topaz, ^d^: Estelite^®^ Σ Quick Sigma, SD: Standard Deviation.

**Table 3:** Intragroup cytotoxicity effect of CRs on hGF and hPDLF cell lines at 24 h and one week time periods.

Cells	CRs	Time Median Min–Max (Mean ± SD)		*p* *
		24 h	One Week	
Cytotoxicity of hGF%	Control	0.00 0.00–0.00 (0.00 ± 0.00)	0.00 0.00–0.00 (0.00 ± 0.00)	1.00
	AF	20.95 −38.09–30.20 (4.53 ± 32.77)	−69.44 −488.88–1.09 (−123.77 ± 183.63)	0.11
	CT	67.29 39.92–78.82 (62.93 ± 16.58)	101.78 48.61–655.55 (202.67 ± 228.76)	0.04
	EQS	46.14 15.01–48.78 (37.12 ± 15.75)	17.36 416–55.55 (20.69 ± 19.02)	0.34
Cytotoxicity of hPDLF%	Control	0.00 0.00–0.00 (0.00 ± 0.00)	0.00 0.00–0.00 (0.00 ± 0.00)	1.00
	AF	−28.73 −50.00–−18.96 (−21.13 ± 10.66)	6.17 1.76–12.29 (7.02 ± 4.06)	0.02
	CT	−22.91 −38.18–−1.56 (−21.23 ± 12.25)	31.02 13.27–37.90 (27.10 ± 9.88)	0.02
	EQS	−44.44 −123.63–−20.31 (−60.70 ± 40.76)	2.86 −6.36–6.89 (1.97 ± 5.29)	0.04

* Wicoxon test, hGF: Human Gingival Fibroblast, hPDLF: Human Periodontal Ligament Fibroblast, AF: Admira^®^ Fusion, CT: Charizma^®^ Topaz, EQS: Estelite^®^ Σ Quick Sigma, SD: Standard Deviation.

**Table 4:** Intragroup viability effect of CRs on hGF and hPDLF cell lines at 24 h and one week time periods.

Cells	CRs	Time Median Min–Max (Mean ± SD)		*p* *
		24 h	One Week	
Viability of hGF%	Control	100 100–100 (100 ± 0.00)	100 100–100 (100 ± 0.00)	1.00
	AF	79.04 69.79–138.09 (95.46 ± 32.77)	169.44 98.90–588.88 (223.77 ± 83.63)	0.11
	CT	67.29 39.92–78.82 (62.93 ± 16.58)	101.78 48.61–655.55 (202.67 ± 228.76)	0.04
	EQS	46.14 15.01–48.78 (37.12 ± 15.75)	17.36 4.16–55.55 (20.69 ± 19.02)	0.34
Viability of hPDLF%	Control	100 100–100 (100 ± 0.00)	100 100–100 (100 ± 0.00)	1.00
	AF	128.73 118.96–150.00 (130.62 ± 10.66)	93.82 87.70–98.73 (92.97 ± 4.06)	0.02
	CT	122.91 101.56–138.18 (121.23 ± 12.25)	68.97 62.09–86.72 (72.89 ± 9.88)	0.02
	EQS	144.44 120.31–223.63 (160.70 ± 40.76)	97.13 93.10–106.36 (98.02 ± 5.29)	0.04

* Wilcoxon test, *p* ˂ 0.05, hGF: Human Gingival Fibroblast, hPDLF: Human Periodontal Ligament Fibroblast, AF: Admira^®^ Fusion, CT: Charizma^®^ Topaz, EQS: Estelite^®^ Σ Quick Sigma, SD: Standard Deviation.
